# Gene Editing in Human Lymphoid Cells: Role for Donor DNA, Type of Genomic Nuclease and Cell Selection Method

**DOI:** 10.3390/v9110325

**Published:** 2017-11-02

**Authors:** Anastasia Zotova, Elena Lopatukhina, Alexander Filatov, Musa Khaitov, Dmitriy Mazurov

**Affiliations:** 1 Faculty of Biology, Lomonosov Moscow State University, 1–12 Leninskie Gory, 119991 Moscow, Russia; ashunaeva@gmail.com (A.Z.); helena.lopatukhina@mail.ru (E.L.); 2 NRC Institute of Immunology FMBA of Russia, 24 Kashirskoe shosse, 115472 Moscow, Russia; avfilat@yandex.ru (A.F.); mr.khaitov@nrcii.ru (M.K.); 3 Cell and Gene Technology Group, Institute of Gene Biology RAS, 34/5 Vavilova Street, 119334 Moscow, Russia

**Keywords:** CRISPR-Cas9, ZFN, gene editing, HIV-1, T lymphocytes, knockout, knockin, cell sorting, cell functionality

## Abstract

Programmable endonucleases introduce DNA breaks at specific sites, which are repaired by non-homologous end joining (NHEJ) or homology recombination (HDR). Genome editing in human lymphoid cells is challenging as these difficult-to-transfect cells may also inefficiently repair DNA by HDR. Here, we estimated efficiencies and dynamics of knockout (KO) and knockin (KI) generation in human T and B cell lines depending on repair template, target loci and types of genomic endonucleases. Using zinc finger nuclease (ZFN), we have engineered Jurkat and CEM cells with the 8.2 kb human immunodeficiency virus type 1 (HIV-1) ∆Env genome integrated at the adeno-associated virus integration site 1 (AAVS1) locus that stably produce virus particles and mediate infection upon transfection with helper vectors. Knockouts generated by ZFN or clustered regularly interspaced short palindromic repeats (CRISPR/Cas9) double nicking techniques were comparably efficient in lymphoid cells. However, unlike polyclonal sorted cells, gene-edited cells selected by cloning exerted tremendous deviations in functionality as estimated by replication of HIV-1 and human T cell leukemia virus type 1 (HTLV-1) in these cells. Notably, the recently reported high-fidelity eCas9 1.1 when combined to the nickase mutation displayed gene-dependent decrease in on-target activity. Thus, the balance between off-target effects and on-target efficiency of nucleases, as well as choice of the optimal method of edited cell selection should be taken into account for proper gene function validation in lymphoid cells.

## 1. Introduction

Targeting DNA with programmable endonucleases opens up an era of functional genomic studies and holds a great promise for therapeutic applications. A DNA double-strand break (DSB) introduced by a specific nuclease is either repaired by non-homologous end joining (NHEJ) machinery to produce a gene knockout (KO) or precisely replaced with a donor DNA by homology recombination (HDR) to generate a knockin (KI). The first programmable DNA-binding proteins including zinc finger nucleases (ZFNs) [1,2] and transcription activator–like effector nucleases (TALENs) [3,4] were not easily reprogrammed or assembled. A technological revolution occurred when the bacterial nuclease Cas9 associated with the clustered regularly interspaced short palindromic repeats (CRISPR-Cas9) guided by a short sequence-specific guide RNA (sgRNA) was demonstrated to efficiently edit the human genome [5,6]. Since then, a wide variety of CRISPR-Cas9 applications for genome manipulations have been reported (reviewed in [7]).

Gene editing in human lymphoid cells is challenging as these cells are difficult to transfect, are less transcriptionally active and have compact chromatin. Hence, chromatin accessibility for ZFN, TALEN or CRISPR/Cas9-sgRNA complex binding in these cells can be limited, while DNA repair may be inadequate. All of this may significantly lower gene targeting either for knocking out or for transgene integration. Nevertheless, the interest in gene surgery in lymphocytes is intense by the necessity to eradicate human immunodeficiency virus (HIV) and cure inherited immunodeficiencies. Some progress has been made in this field. For example, treatment of CD4^+^ lymphocytes or their progenitor cells with ZFN, TALENs, or CRISPR/Cas9 designed to knock out the C-C chemokine receptor type 5 (CCR5) coreceptor of HIV was demonstrated to effectively protect cells from HIV-1 infection in vitro [8,9,10,11], in mouse model [8], or in clinical trials [12] (Sangamo Bioscience reports). The chimeric antigen receptor T cells engineered with TALENs have been successfully applied to treat leukemia (Leyla’s phenomenon) [13], while autologous tumor-associated T cells treated with CRISPR to knockout *programmed cell death protein 1* (*PD-1*) gene have been recently administered to patients in China to combat lung cancer.

In this study, we aimed to comparatively examine the efficiencies of KI and KO generation in human lymphoid and non-lymphoid cell lines using ZFN, CRISPR/Cas9 and its mutant modifications, varying target loci or DNA donor templates. Using ZFN targeting the human adeno-associated virus integration site 1 (AAVS1) locus, we have been able to engineer Jurkat and CEM cells with an HIV-1 ∆Env genome containing 1 kb deletion in 3′-portion of *env* gene. Despite the potential of negative effect of viral protein expression, isolated T cell clones could be expanded and used for large-scale HIV-1 virion production or quantitation of HIV-1 replication when cotransfected with the viral helper plasmids. We also drew attention to the methods of gene-edited cell selection showing that cell clones exerted biased functionality relative to polyclonal cells isolated by fluorescence-activated cell sorting (FACS). The design of sgRNA specific to different targets always resulted in an efficient gene inactivation, though with varying efficiencies. In order to maximize the fidelity of Cas9, we combined the eCas9 1.1 modification [14] with the nickase mutation D10A [15]. While the resulting eCas9n performed as well as Cas9n in a model of *green fluorescent protein turbo* (*GFPt*)-reparation in 293T cells, its on-target activity in CEM T cells for all tested genes was severely reduced. Thus, gene editing in lymphoid cells is a challenging but achievable goal with a potential to knock in large fragments of DNA and limitations posed by the less efficient gene-editing component delivery, and, as a consequence, low KO and especially low KI rates.

## 2. Materials and Methods

### 2.1. Cell Cultures and Reagents

The human CD4 T cell lines Jurkat E6-1 and CCRF-CEM, the monocytic cell line U937, and the human embryonic kidney (HEK) 293T cells were obtained from American Type Culture Collection (ATCC) (Mannasas, VA, USA). The human Raji/CD4 B cell line was a gift from Vineet N. KewalRamani (NCI at Frederick, MD, USA). All suspension cells were maintained in Roswell Park Memorial Institute (RPMI) 1640 culture medium supplemented with 10% fetal bovine serum, 2 mM l-glutamine, and 40 μg/mL gentamicin. To select for CD4 expression, 400 µg/mL Geneticin (Gibco, Gaithersburg, MD, USA) was added to the culture of Raji cells. HEK 293T cells were grown in Dulbecco’s modified Eagle medium (DMEM) culture medium containing 10% fetal bovine serum, 50 µM β-mercaptoethanol, 2 mM l-glutamine, and 40 μg/mL gentamicin. The primary anti-human CD mouse monoclonal antibodies (mAbs) against CD4 (clone EM4), CD45 (clone LT45), CD54 (clone TD4D9), and CD82 (clone MC8D12) were all obtained from Sorbent (Moscow, Russia); mAbs specific to CD18 (clone MEM-48) and CD50 (clone MEM-171) were obtained from Exbio (Vestec, Czech Republic). The anti-human CCR5 mAb was from BD Pharmingen (San Diego, CA, USA). The primary anti-CXCR4 mAb (clone 12G5), and the secondary goat anti-mouse Ab conjugated to phycoerythrin (PE) were purchased from Santa Cruz (Dallas, TX, USA).

### 2.2. Plasmids and Donor DNAs

The plasmid encoding AAVS1-specific ZFN under control of cytomegalovirus (CMV) promoter pCMV-ZFN-AAVS1 was constructed by cloning a 2.1 kb DNA fragment designed to encode left and right ZF proteins fused to FokI heterodimer and separated by a 2A peptide sequence (see Figure S1) into mammalian expression vector pCMVpA (Addgene, Cambridge, MA, USA) using PstI/KpnI restriction sites. The left and right ZF sequences were flanked by EcoRI/BamHI and XbaI//NheI restriction sites, respectively, so that they could be easily replaced to reprogram the nuclease. ZF sequences were assembled by overlapping PCR using conserved DNA oligos and oligos specific for either the AAVS1 [16] or CCR5 [8] or CXCR4 [17] locus (Figure S2 and Table S1). To generate AAVS1 donor vectors with an appropriate polylinker, the left and right arms of homologies were PCR-amplified from pZDonor-AAVS1 vector (Sigma, St. Louis, MO, USA) using pairs of primers listed in Table S2, and cloned into pBluescript II KS (+) vector (Stratagen, La Jolla, CA, USA) at SacII/EcoRI and EcoRI/KpnI restriction sites, respectively. Then, the SV40 polyA signal from the pCMVpA plasmid was PCR-amplified and cloned between homology arms at EcoRI/XhoI sites. To generate the donor vector pAAVS1-∆8.2R containing the NL4-3 HIV-1 Env-genome, we introduced a Mlu I restriction site upstream of the CMV promoter in the pCMV-∆8.2R plasmid (Addgene, Cambridge, MA, USA) using Quick Change PCR Mutagenesis with the primers specified in Table S2. Afterwards, the entire 8.2 kb DNA fragment between the MluI and BstEII sites encoding HIV-1 was subcloned into the pAAVS1 donor vector upstream of SV40pA at MluI/EcoRI sites (BstEII and EcoRI restriction sites were blunted with Klenov DNA polymerase). The Tag blue fluorescent proteins (Tag-BFP) expression cassette was cloned into the pAAVS1 donor vector in a reverse orientation relative to the homology arms by subsequent cloning of the SV40pA and Tag-BFP DNAs that had been PCR-amplified from the pCMVpA and pTagBFP-C plasmids (Evrogen, Moscow, Russia), respectively, using primers and restriction sites specified in Table S3. Finally, the CMV promoter from the pCMV-Tet3G vector (Clontech, Mountain View, CA, USA) was subcloned into the donor vector at EcoRI/XhoI restriction sites. The sgRNA expressing vector pKS gRNA BB, the plasmids for the expression of wild-type Cas9 (Addgene #41815), and its nickase mutant D10A were described earlier [18]. The expression plasmids for the low off-target versions of Cas9 (pcDNA 3.3-eCas9 and pcDNA 3.3-eCas9n) were generated by introducing three mutations (K848A, K1003A, and R1060A [14]) into the Cas9 sequence. To that end, a BamHI/XhoI fragment of Cas9 DNA was temporarily cloned into the pBluescript II KS (+) vector for Quick Change Mutagenesis with respective primers (Table S4). After sequence verification, the mutated fragment was cloned back into the pcDNA 3.3-TOPO vector. The gRNA target sequences for the double-nicking technique were selected using a web-based resource http://tools.genome-engineering.org (Table S5), and designed for cloning into pKS gRNA BB vector via a BbsI restriction site. The lentiviral vector pGIPZ (Open Biosystems) with a one-nucleotide deletion mutation at the beginning of the GFP-turbo ORF (C57del) was generated by Quick Change PCR mutagenesis (primers are indicated in Table S6) on an XbaI/NotI fragment of GFP-turbo DNA that had been temporarily cloned into pBluescript II KS (+) vector. After sequence verification, the mutated DNA fragment was cloned back into pGIPZ plasmid. The target sequences of GFP-turbo (GFPt) specific sgRNAs and respective oligonucleotide donor DNAs are listed in Table S5. A protospacer adjacent motif (PAM)-mutated PCR-donor for the *GFPt* gene was generated by a standard overlapping PCR with four oligos indicated at the bottom of Table S6. All plasmids generated here were sequence-verified.

### 2.3. Transfections and Infections

The human 293T cells were transiently transfected using Lipofectamine 2000 (Invitrogen, Carlsbad, CA, USA) according to the manufacturer’s instructions. The lymphoid cell lines CEM and Raji/CD4 were transfected using Neon electroporation system (Invitrogen) by a single 30-ms pulse at 1350 V; the Jurkat and U937 cells were electroporated by three 10-ms pulses at 1450 V and 1400 V, respectively. To generate cell lines stably expressing the mutated GFP-turbo, 293T cells grown in a 10-cm dish were cotransfected with 4 µg of pCMV-∆8.2R, 6 µg of pGIPZmut, and 1 µg of pCMV-VSVG plasmid DNA expressing protein G from vesicular stomatitis virus. The next day, the medium was replaced, and virus-like particles (VLPs) were harvested and used to infect 293T cells (with a low dose) or CEM cells (with a high dose). Three days postinfection, the transduced cells were selected by growing in the presence of puromycin (Sigma, St. Louis, MO, USA) at a concentration increasing from 0.4 to 1.0 µg/mL. The cell coculture infections were performed as described earlier [19,20]. Briefly, to set up HIV-1 infection, 10^6^ CEM cells were electroporated with 3 µg of pUCHR-inLuc-mR vector DNA, 2 µg of pCMV∆8.2R plasmid DNA, and 0.8 µg of the pIIINL-4env plasmid, which expresses Env from HIV-1 strain NL4-3. To initiate HTLV-1 infection, cells were cotransfected with 3 µg of pCRU5-inLuc-mR vector DNA, and 2 µg of pCMVHT1-M plasmid DNA. The transfected cells were mixed immediately with 10^6^ Raji/CD4 or Raji/CD4-TagBFP target cells. Sixteen hours prior to harvesting, cells were stimulated with 20 nM PMA to enhance reporter expression. Cells were collected 72 h after cell coculture initiation, and extracted with Glo lysis buffer (Promega, Madison, WI, USA), and luciferase (Luc) activity was measured by using Promega luciferase reagent and a Glomax 20/20 Luminometer instrument (Promega, Madison, WI, USA).

### 2.4. KO and KI Generation, Clonal Selection and Detection of Transgene Integration

To generate Jurkat and CEM cells with a stable isogenic integration of HIV-1 packaging vector, 10^6^ cells were electroporated with 5 µg of pCMV-ZFN-AAVS1 and 5 µg of pAAVS1-∆8.2R plasmid DNAs. The next day, cells were single-cell-cloned in six 96-well plates and grown for about two weeks. The supernatants from the obtained clones were then harvested and quantified for the viral Gag expression using an HIV-1 p24 ELISA Kit (VectorBEST, Novosibirsk, Russia). The Raji/CD4 cells with the Tag-BFP expression from the AAVS1 locus were obtained as described above except that the donor vector was pAAVS1-TagBFP, and the selection of clones or polyclonal cells was performed using a FACS cell sorter (see below). The BFP^+^ or ∆8.2R^+^ cell clones were expanded, and the correctness of transgene integration was estimated using a standard PCR, which was set up with 200 ng of genomic DNA per reaction and the pairs of primers listed in Table S7. To estimate CRISPR/Cas9-mediated KI, the 293T-GFPt-mut cells grown in a 12-well plate were cotransfected with 0.25 µg of sgRNA expression plasmid (if two sgRNAs were used for double nicking (DN), than with 0.125 µg of each one), 0.75 µg of plasmid encoding wild-type Cas9 (or a mutant form as indicated), and 0.5 µg of donor DNA. After 6 h, the medium was changed, cells were grown overnight and then split. Cells were trypsinized and analyzed for GFPt fluorescence 72 h posttransfection. For gene editing in lymphoid cells, 10^6^ suspension cells were electroporated with a pair of sgRNA plasmids (0.5 µg of each one), 3 µg of nickase (or another mutant) variant of Cas9 expression plasmid, and, if required, 1 µg of donor DNA. Cells were cultured for 72 h or longer as indicated in Results, and analyzed for GFPt or cell surface antigen expression by FACS.

### 2.5. Immunofluorescence, Flow Cytometry and Cell Sorting

For immunostaining of surface antigens, cells (10^6^) were incubated in phosphate-buffered saline (PBS) containing 0.1% sodium azide, 0.5% bovine serum albumin (BSA), and 5 µg/mL primary mAb for 30 min on ice. Cells were washed twice with PBS, incubated with the anti-mouse PE-labeled Ab diluted as indicated for the primary Ab for another 30 min at +4 °C, and then washed again with PBS. Cells pelleted after immunostaining or unstained cells (GFPt expression) were resuspended in PBS and analyzed using a Cytoflex S (Beckman Coulter, Brea, CA, USA) flow cytometry instrument. GFPt and PE signals were detected with the 488-nm and 561-nm lasers, respectively. Raji/CD4 cells transgenic for Tag-BFP protein were either single-cell-sorted into 96-well plates or pooled in a single tube using a FACSAria II Instrument (Becton Dickinson Biosciences, San Jose, CA, USA) and 408-nm laser excitation of Tag-BFP. The collected data were analyzed by CytExpert and then presented using FlowJo, LLC software (Ashland, OR, USA).

### 2.6. Data/Materials Availability

Two plasmids described in the manuscript, pAAVS1-∆8.2R and pCMV-ZFN-AAVS1, have been deposited at Addgene under accession numbers 89,706 and 89,707. The other constructs and dataset generated during this study are available from the corresponding author upon request.

## 3. Results

### 3.1. T Cell Lines Can Stably Express HIV-1 Packaging Genome Integrated into the AAVS1 Locus

**Figure 1:**
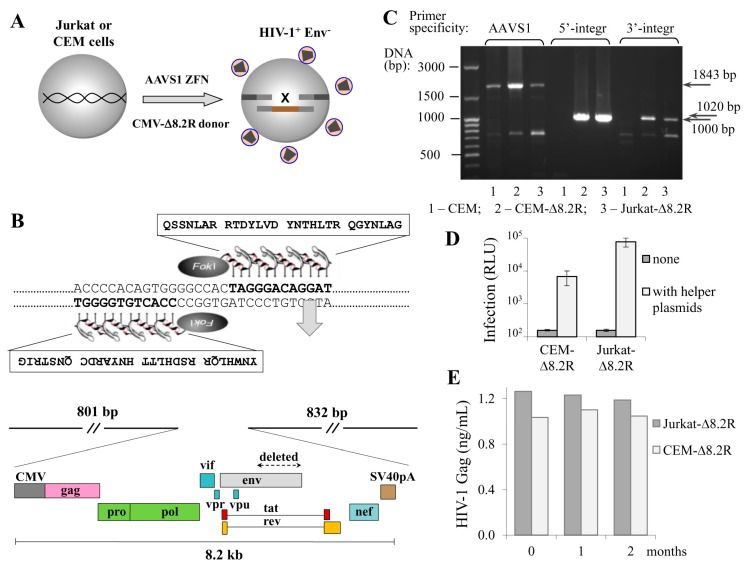
Engineering human T cell lines with the human immunodeficiency virus type 1 (HIV-1) packaging genome integrated into adeno-associated virus integration site 1 (AAVS1) locus. (**A**) a schematic drawing showing that cotransfection of CEM or Jurkat T cells with the AAVS1-specific zinc finger nuclease (ZFN) expression plasmid and HIV-1 donor DNA ∆8.2R resulted in generation of cells producing nascent virus-like particles; (**B**) the amino acid sequences of AAVS1-specific zinc finger proteins that recognized the DNA target sequences (on the top) and a schematic map of pAAVS1-∆8.2R donor vector (on the bottom) used to generate HIV-1 transgenic T cells; (**C**) confirmation of ∆8.2R transgene integration at the AAVS1 locus. Genomic DNAs from CEM cells (negative control), CEM-∆8.2R, and Jurkat-∆8.2R clones (cells containing the transgene) were isolated and used to set up PCR with integration-specific primers; (**D**) transgenic T cells could mediate HIV-1 infection. Cells were transfected with a mock plasmid (grey bars) or pUCHR-inLuc-mR and pIII-NL4 Env plasmid DNAs (open bars) and mixed with Raji/CD4 target cells. Luciferase activity was measured 3 d posttransfection. The averages of three independent experiments with the standard deviations are presented; (**E**) stability of nascent virion production by transgenic cells. Supernatants from freshly generated CEM-∆8.2R and Jurkat-∆8.2R cells (zero time point) or after 1 or 2 months of passage were harvested and quantified for viral Gag production using p24 ELISA Kit.

The transduction of cells with lentiviral vectors remains the most efficient and popular method of ectopic gene expression in a wide variety of cell types. However, the method is limited by the length of the packaging RNA: when it is longer than 6 kb, the titer of lentiviral particles falls dramatically and the more difficult-to-transduce cells, such as primary cells or lymphoid cell lines, cannot be successfully infected. Moreover, the lentiviral delivery of HIV genome is complicated by viral signal overlaps. Here, using ZFN, we aimed to deliver the HIV-1 packaging genome to the human safe harbor locus AAVS1, where transgenes are not silenced and generally do not disturb the regulation of neighboring genes [21]. Based on the published sequence of AAVS1-specific zinc fingers [16], we designed an expression vector for AAVS1-ZFN that can be easily reprogrammed to express ZFN with different specificities using overlapping PCR and site-specific restrictases (see Methods section, Figures S1 and S2, and Table S1 for details). Although the using of longer homology arms (HAs) results in higher rates of HDR, we limited the length of the AAVS1-HAs to 800–900 bp, as long HAs complicate PCR-based genotyping of targeted alleles and substantially increase the donor plasmid size. The AAVS1-HAs were assembled into a small plasmid pBluescript II KS (+), and an 8.2-kb DNA encoding ∆Env HIV-1 from the NL4-3 strain under control of the CMV promoter was cloned between the HAs. The obtained ZFN and donor plasmids were cotransfected into Jurkat and CEM T cell lines to generate transgenic cells that stably produce nascent viral particles. Figure 1A schematically illustrates this process, and Figure 1B shows the key elements of the AAVS1-specific ZFN and donor vectors. Transfected cells were single-cell-cloned into six 96-well plates (for each cell culture), and growing clones were tested for viral particle production using HIV-1 p24 ELISA Kit. Four out of approximately 300 CEM clones, and one of 150 Jurkat clones were positive for p24 expression. The positive clones were recloned and retested for virus production to confirm cell clonality. Next, we performed PCR with integration-specific primers to analyze the specificity of target integration, and found that two CEM clones among the total five selected clones were negative in PCR with both 5′- and 3′-integration-specific primers. This suggests that about 40% of HIV-1 integration events occurred outside the AAVS1 locus, while the frequency of target-specific integration of HIV-1 genome calculated relative to all transfected and cloned cells was about 0.5–1%. Finally, we selected one CEM clone and one Jurkat clone with the highest growth rate, and named them CEM-∆8.2R and Jurkat-∆8.2R, respectively. Apparently, these two clones had mono-allelic integration of the ∆8.2R transgene, as intact allele(s) were detected in these cells by PCR with AAVS1-speicific primers (Figure 1C, left three lines). Since bi-allelic integration is a less frequent event, it was not a surprise that we did not detect it among the clones we had tested. To determine whether the generated transgenic cells could mediate HIV-1 infection, we cotransfected them with two helper plasmids, i.e., inLuc transfer vector and HIV-1 Env expression plasmid, and mixed with Raji/CD4 target cells to set up coculture infection [19]. As demonstrated in Figure 1D, in the absence of helper plasmids the luciferase activity was undetectable. By contrast, transfecting helper plasmids into either CEM-∆8.2R or Jurkat-∆8.2R cells resulted in decent levels of luciferase activity, though infection mediated by Jurkat-∆8.2R cells was one order of magnitude higher than that mediated by CEM-∆8.2R cells. Next, using p24 ELISA we measured dynamics of HIV-1 viral particle production by the generated cells, and showed that it was stable for at least two months (Figure 1E). Together, these results suggest that HIV-1 ∆Env genome integrated at the AAVS1 locus of the engineered CEM-∆8.2R and Jurkat-∆8.2R cells is not silenced epigenetically as it frequently occurs after natural integration of HIV, and that the produced virus-like particles are infectious upon pseudotyping with viral Env.

### 3.2. Functional Biases of Gene-Edited Cells: Clones versus Pooled Population

**Figure 2:**
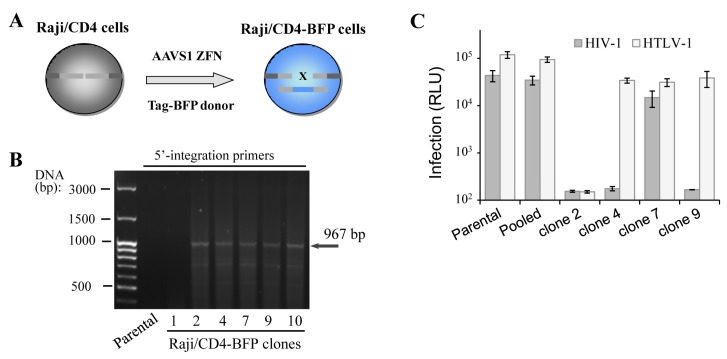
Functionality of gene-edited cells selected via cloning or sorted as pooled population. (**A**) schematic representation of Raji/CD4 cell generation with Tag blue fluorescent protein (Tag-BFP) engineered using ZFN; (**B**) PCR with 5′-integration-specific primers to determine whether the integration of the Tag-BFP expression cassette at the AAVS1 locus of isolated Raji/CD4 clones is correct; (**C**) quantification of HIV-1 and HTLV-1 cell coculture levels of infection in Tag-BFP clones and in the sorted population. CEM cells were transfected to initiate HIV-1 or HTLV-1 infection as outlined in Methods, and mixed with the parental Raji/CD4 cells (control) or Tag-BFP^+^ cells selected as a pool or clones. Three days later, cells were lysed, and the levels of infection were estimated by measuring the levels of luciferase activity. Data are representative of at least three independent experiments.

In spite of successful isogenic integration of HIV-1 genome in T cells, the engineered clones were characterized by low growth rate, high sensitivity to electroporation-caused cell death and lower capacity to mediate HIV-1 infection when compared to parental cells. The observed side effects could be explained by accumulation of mutations generated by the off-target activity of genomic nuclease and/or by negative effects of some viral proteins, for instance Vpr and Vif, on cell replication and viability. To exclude viral influence on cell behavior, we generated Raji/CD4 cells with a Tag-BFP expression cassette integrated at the AAVS1 locus (Figure 2A), assuming that the expression of the fluorescent protein should not affect cell function. We reasoned that in a polyclonal BFP-positive population, cells with the least off-target genome damage should outgrow cells with severe negative off-target effects, which would be impossible with clones. To pursue this idea, two weeks after transfection with ZFN and donor plasmid DNAs, when the transient expression of Tag-BFP dies down, Raji/CD4 cells were either single-cell-cloned or isolated as a polyclonal population using a FACS sorting instrument. Six generated BFP^+^ clones were selected and tested for transgene integration into the AAVS1 locus using PCR and 5′-integration-specific primers (Figure 2B). Five out of six clones displayed locus-specific integration, i.e., the HDR rate was >80%, which is substantially higher than the integration rate for HIV-1 ∆Env (see above). This suggested that shorter transgenes integrate not only more frequently but also more specifically than longer constructs. To compare functionality of the generated clones versus polyclonal cells, we set up two retroviral infections, HIV-1 and HTLV-1, where Raji/CD4 cells were used as target cells and transfected CEM cells were viral effectors (see details in the Methods section). Replication of these viruses is highly dependent on cellular factors, some of which are required for both viruses, but the others are specific for only one, suggesting that these viral replication tests can serve as a good tool to roughly estimate cellular functionality. The infection tests (Figure 2C) demonstrated that two clones did not respond to HIV-1, but responded to HTLV-1 infection; one clone was resistant to both retroviral infections, and only one clone was infected with both viruses, although at a level lower than that measured in parental cells. In contrast to clonally selected cells, the pool of sorted BFP^+^ cells demonstrated replication of HIV-1 and HTLV-1 at the levels statistically not different from the levels of infection detected for parental cells. Thus, although gene-edited cells sorted as pooled polyclonal populations could be heterogeneous harboring off-target mutations and responding differently to stimuli, they displayed much better functional characteristics in our viral tests than clones.

### 3.3. Quantifying Knockin Rates in Lymphoid and Non-Lymphoid Cells Using Different Donor DNAs

**Figure 3:**
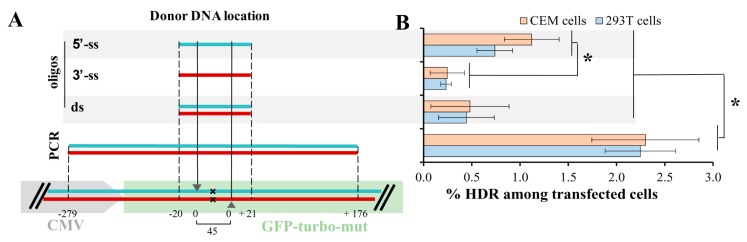
Knockin rates in 293T and CEM cells detected with different donor DNAs. (**A**) positioning single-strand (ss) and double-strand (ds) donor DNAs (flanked by dotted lines) relative to cleavage sites (shown by arrows) produced by Cas9 nickase in the mutated (x) *gfp-turbo* gene. The color of donor DNA strand (blue is coding and red is non-coding) matches the color of targeted gene strand; (**B**) the homology directed repair (HDR) rates in 293T-GFPt-mut and CEM-GFPt-mut cells were estimated at day 3 after transfection with plasmid DNAs encoding Cas9n, a pair of green fluorescent protein (GFP)-turbo specific gRNAs and indicated donor DNA. The proportion of GFP-positive (repaired) cells relative to all cells was normalized to the level of transfection, measured in each individual experiment. The results from at least three independent experiments are presented as average values with the standard deviations. * Data are statistically different at *p* < 0.05.

With ZFN, we approximately estimated KI levels for Tag-BFP and ∆8.2R transgenes. It has been reported that the type of donor DNA (i.e., single-stranded (ss), double-stranded (ds), asymmetric) as well as the length of donor DNA [22,23] can either increase or decrease the level of HDR. While the influence of donor DNA on the level of HDR has been widely studied using wild-type Cas9, this was not comprehensively examined when the DNA DSB was induced by double nicking [15]. To quantify the levels of HDR in lymphoid and non-lymphoid cells, we established a GFPt-mut cell model, where a cytosine at the position 37 from the start-codon of *gfp-turbo* gene was deleted resulting in the formation of a premature stop-codon. Two sgRNAs targeting the *gfp-mut* gene upstream and downstream of the mutation site, and 4 types of donor repair DNAs (5′-ss, 3′-ss, ds-oligos, and a 500-bp PCR product, all with nonsense PAM-inactivating mutations) were designed and generated (see Methods for details). Figure 3A schematically illustrates the positions of donor DNAs relative to the deleted nucleotide (x) and DNA cut sites (arrowheads) in the *gfp-turbo* gene. CEM and 293T cell lines were transduced with a lentiviral vector encoding the mutated *gfp-turbo* gene and then selected with puromycin. The obtained CEM-GFPt-mut and 293T-GFPt-mut cells were cotransfected with a pair of GFP-specific sgRNAs, Cas9n, and donor DNA. Three days posttransfection the levels of HDR were estimated as percentages of cells expressing GFPt. Since the efficiency of transfection for 293T cells exceeded that obtained for CEM cells, we normalized the levels of HDR to the transfection efficiencies with GFP expression plasmid, which were measured individually for every experiment. The HDR levels with all tested donor DNAs measured in 293T cells were higher than HDR levels detected in CEM cells, but were not different after normalization to the levels of transfection (Figure 3B). The 500 bp PCR-donor mediated HDR was at least twofold better than any of ss- or ds-oligonucleotide donors. Interestingly, the 5′-ss oligonucleotide donor, which was complementary to the target strand of DNA for the upstream sgRNA and to the non-target strand for the downstream sgRNA, was more efficient in HDR than 3′-ss oligonucleotide donor with the opposite complementarity. This suggested that the process of DNA strand cut and release during double nicking could be separated in time. Possibly, the downstream nicked strand was released first, which favored 5′-ss donor invasion over 3′-ss donor invasion. This idea is consistent with experimental and molecular structure data reported for wild-type Cas9 DSB repaired by ss-oligonucleotides [22]. In comparison to the 5′-ss donor or long PCR donor, the short ds-oligonucleotide donor was less potent in repairing GFPt, which is similar to the results reported for wild-type Cas9. In summary, the HDR rates detected in the lymphoid cell line CEM were similar to those measured in the non-lymphoid HEK 293T cells, when the efficiency of CRISPR/Cas9 components delivery is taken into account. Similar to the situation with blunt-end DNA DSBs induced by the wild-type Cas9, the length and type (5′-ss, 3′-ss or ds) of donor DNA substantially influence the repair of DNA DSBs with long overhang ends generated by the Cas9 nickase; this should be taken into account when designing donor templates for the DN method.

### 3.4. Evaluation of ZFN and Cas9n Mediated Gene Knockout Rates in Hematopoietic Cell Lines

**Figure 4:**
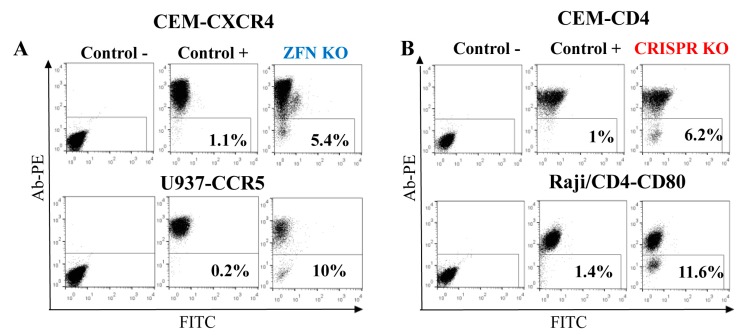
Efficiency of gene KO with ZFN (**A**) and CRISPR/Cas9 (**B**) in lymphoid and myeloid cell lines. Indicated cell lines were transfected with either ZFN expression plasmid DNA or two juxtaposed sgRNAs plus Cas9n expression vector DNAs. After a week, cells were surface-stained with corresponding primary mAbs and secondary anti-mouse PE-labeled Ab, and analyzed by flow cytometry. As a negative control, the primary mAb was omitted. As a positive control, immunostained non-transfected parental cells were used. Typical flow cytometry dot plots are shown.

As shown above, gene-edited cells were more functionally relevant when FACS-sorted as polyclonal populations. Therefore, to evaluate KO efficiency in lymphoid cells, we focused on genes that encode proteins expressed on the cell surface. This made KO cell analysis and sorting easy, requiring only staining with respective antibodies. In order to estimate the rates of ZFN- and Cas9-mediated gene KO, we first generated ZFN expression plasmids to target *cxcr4* and *ccr5* genes by replacing DNA encoding the AAVS1 DNA-binding domains of ZF proteins with DNAs encoding ZFs specific for CXCR4 [17] or CCR5 [8] (see details in the Methods section). Then, using http://tools.genome-engineering.org web resource, we designed pairs of sgRNAs for DN [15] that targeted human CD4 and CD80 genes. The DN technique was selected because Cas9n has been proved to generate much less off-target effects than the wild-type Cas9 [15]. In addition, 5 μg of ZFN expression plasmid or 3 μg of Cas9n expression plasmid with 1 μg of each two sgRNA-encoding plasmids were transfected into the different human suspension cell lines to generate KOs. The choice of gene targeting techniques and of the cell lines was based on the accessibility of ZF sequences and on the level of target protein expression on the cell surface. The efficiencies of gene KO were quantified seven days post-transfection as percentages of negative cells among all cells after surface staining with appropriate antibodies. As shown in Figure 4, the levels of KO generated via ZFN, particularly CXCR4 KO in CEM T cells and CCR5 KO in the U937 monocytoid cell line (A), were comparable to the efficiencies of KO obtained with Cas9n for CD4 in CEM cells and for CD80 in Raji/CD4 B cells (B), and varied from 5 to 12%. Thus, both nucleases were efficient at generating KO in hematopoietic cell lines, suggesting that despite the different mechanisms of DNA recognition, ZFN and Cas9 could easily access chromatin for editing different genes.

### 3.5. Dynamics and Efficiency of Gene KO with Cas9n and eCas9n

**Figure 5:**
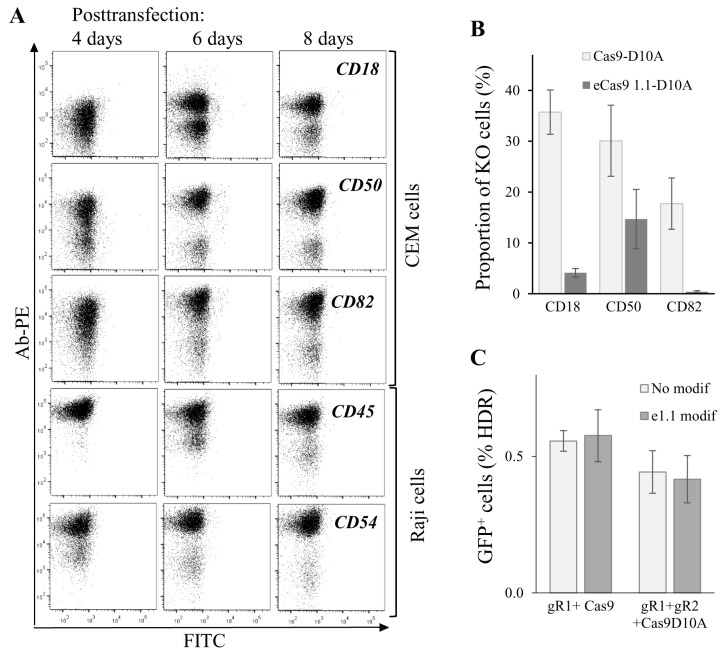
Dynamics and efficiencies of knockout (KO) phenotype development generated for the various genes and with different CRISPR/Cas9 methods. (**A**) dynamics of KO phenotype development tested for different genes. CEM or Raji/CD4 cells were transfected to knockout indicated genes using the double nicking method. At different time points after transfection, cells were immunostained with the appropriate primary mAb and the secondary PE-labeled Ab, and then analyzed by flow cytometry. Typical dot plots are shown; (**B**) comparing on-target activities of Cas9n (Cas9-D10A) with those of eCas9n (eCas91.1-D10A) to knock out three different genes in CEM cells. CEM cells were transfected with a pair of gene-specific sgRNA expression plasmid DNAs and either Cas9n or eCas9n expression vector DNA. In a week, cells were immunostained and analyzed by FACS. The percentages of KO cells averaged from three independent experiments with the standard deviations are shown on histogram; (**C**) both wtCas9 and Cas9n modified with K848A, K1003A, R1060A mutations retain on-target cleavage activity, when *gfp-turbo* gene is targeted. 293T-GFPt-mut cells were cotransfected either with 5′-ss oligonucleotide donor DNA, one downstream sgRNA plasmid DNA targeting GFPt, and indicated Cas9 expression vector (left bars) or with the same donor DNA, a pair of GFPt-specific sgRNAs plasmid DNAs, and plasmid encoding indicated Cas9 nickase (right bars). At day 3 after transfection, samples were analyzed for cells expressing GFPt using flow cytometry. Data are representative of at least three independent experiments.

To further characterize gene editing in lymphoid cells, we measured kinetics of KO phenotype development upon targeting five human genes, CD18, CD50 and CD82 in CEM cells, CD45 and CD54 in Raji/CD4 cells. Cells were cotransfected with Cas9n and two sgRNA expression plasmid DNAs and analyzed for plasma membrane expression of corresponding proteins at different time points after transfection. As shown in Figure 5A, cells with CD50 and CD54 KO phenotypes could be clearly distinguished from the intact population by flow cytometry as early as four days post-transfection. A clear KO phenotype for CD18, CD82, and CD45 genes began to develop at day 6 after transfection. Cells with all tested gene KOs completely lost expression of target proteins on their surface at day 8 posttransfection. The observed variations in phenotype development could be explained by the differences in protein turnover. Nevertheless, we concluded that approximately in a week after transfection, subpopulations with a KO phenotype could be observed for most of the Cas9-targeted genes, and these subpopulations can be selected for further analysis and sorting. Since off-target activity of Cas9 induces genome instability and cell function biases, many efforts have been made to increase the fidelity of Cas9 DNA recognition/processing. We combined two of these reported methods, DN [15] with eCas9 1.1 modification that weakened the nucleolytic activation of Cas9 occurred upon mismatched binding of sgRNA with DNA [14], and estimated on-target activity of modified Cas9. To obtain eCas9n, three mutations (K848A, K1003A, R1060A) were introduced into the pcDNA3.3 Cas9n expression plasmid. The knock-out efficiencies of three human genes, CD18, CD50, and CD82, in CEM cells were compared for Cas9n and eCas9n at day 7 after cell transfection (Figure 5B). Surprisingly, eCas9n demonstrated a significantly lower on-target activity, which varied from a twofold decrease for CD50 to almost no activity for CD82 when compared to Cas9n. Using the model of GFP-turbo repair described above, we additionally tested the wtCas9, Cas9n, eCas9 and eCas9n on-target activities in 293T cells. In contrast to results obtained for endogenous gene KO in CEM cells, in 293T-GFPt-mut cells, the on-target activity of the eCas9 1.1 mutant was not lower than the activity of wild-type Cas9 or Cas9n (Figure 5C). Together, these data suggest that on-target activity of eCas9n varied between genes and could be substantially reduced relative to Cas9n, which should be taken into account when designing gene KO with CRISPR/Cas9.

## 4. Discussion

The efficiency of gene editing in living cells, a critical parameter for successful KO and KI generation, depends not only on the effectiveness of editing tools per se, but also on the methods used to deliver these tools into the cell. The latter issue is important because lentiviral transduction is the most powerful method to deliver CRISPR/Cas9 into many cell types, and is widely used for diverse applications including sgRNA library screens. However, the stability of lentiviral expression raises a serious issue of off-target side effects, which are amplified by prolonged expression of CRISPR components. In addition to efforts made to improve the nuclease fidelity, the transient expression of CRISPR/Cas9 from plasmids, RNAs or ribonucleoprotein complexes transfected into cells may help to avoid off-target effects registered in gene-edited cells [24,25,26,27]. In addition, lentiviruses cannot be used to deliver donor DNA, and, therefore, to edit genome via HDR.

In our work, we used lipofection and electroporation to deliver both the plasmid DNAs, which encoded gene-editing components and the donor DNAs. This allowed us to investigate not only KO, but also KI events in human lymphoid cell lines. Using this approach, we demonstrated that site-specific KI of donor template sized from 8.2 kb to less than a hundred bases was achievable for lymphoid cell lines. Generally, the rates of KO and KI in these cells were similar to those detected in nonlymphoid 293T cells when normalized to transfection efficiency. The generated KI cells were functional; however, if they were selected by cloning, the chance to derive functionally abnormal cells increased substantially. In contrast, sorted polyclonal cells in our tests were nearly as good as unedited cells, as was confirmed by replication of HIV-1 and HTLV-1 in these cells. Thus, targeting genes encoding cell surface molecules for KO or KI represents an easy task, as it can be coupled to the cell sorting or magnetic separation of immunolabeled cells. Unfortunately, this selection technique is not applicable if the targeted gene encodes an intracellular or a secreted protein.

Despite the improvements in the specificity of DNA targeting by CRISPR/Cas9 and other genomic nucleases, the off-target effects remain a major issue that restrict gene editing in clinical practice. We constructed the eCas9n nuclease that combined the nickase mutation D10A with the three mutations that had been reported to increase Cas9 fidelity [14], expecting that this would further minimize off-target activity of Cas9, and evaluated on-target activity of the mutant. In the GFPt-mut restoration model, both eCas9 and eCas9n worked as efficiently as Cas9 or Cas9n. However, on-target activity of eCas9n measured for three endogenous genes in CEM T cells was significantly reduced or nearly abolished relative to eCas9. This indicates that mutations in the positively-charged groove of nuclease could decrease not only off-target, but also on-target activity of Cas9, which seemed to be dependent on target genes.

In future, the generated human transgenic T cell lines producing HIV-1 nascent virus like particles from the packaging vector integrated into the AAVS1 locus can be further engineered to stably express viral Env and the inGFPt-mR reporter [20]. The resulting transgenic HIV-1 effector cells mixed with Raji/CD4-Tag-BFP target cells will quantitatively measure the cell synapse formation and the level of cell coculture infection in one step using multicolor flow cytometry analysis. Due to the clonal nature of the generated transgenic cells and their sensitivity to electroporation, we believe that the finalizing steps of HIV-1 effector cells engineering will be more achievable via lentiviral or retroviral transduction. The transgenic HIV-1 effector cells will be a good instrument to study not only mechanisms of cell-to-cell transmission, or to screen antivirals, but can also help to understand cellular restriction in the context of cell-free and cell-to-cell virus replication.

## 5. Conclusions

We demonstrate that the 8.2 kb HIV-1 ∆Env packaging genome could be integrated into the human AAVS1 locus of T cell lines and stably expressed without antibiotic selection. The gene editing rates detected in human lymphoid cells were similar to those obtained in HEK 293T cells, and were mainly determined by efficiency of transfection. When possible, gene-edited cells should be selected for functional analysis as pooled polyclonal populations, in order to avoid clonal biases. The Cas9n and eCas9n, the latter generated by us, should be preferred for gene editing over the wild-type Cas9 because of fewer off-targets. However, on-target activity of eCas9n could be significantly reduced, and, therefore, should be tested individually for each gene. The recently described high-fidelity SpCas9-HF1 [28] and its combination with eCas9 1.1 called hyper-accurate HypaCas9 [29] retained high on-target activity, offering another tools to solve off-target problems.

## Supplementary Materials

The following information is available online at www.mdpi.com/1999-4915/9/11/325/s1, Figure S1: sequence of AAVS1-specific ZFN module, Figure S2: strategy to assemble ZFP coding DNA using overlapping PCR, Table S1: the list of primers used to change the specificity of ZFN, Table S2: primers for engineering pAAVS1-∆8.2R donor plasmid, Table S3: primers for engineering pAAVS1-TagBFP donor plasmid, Table S4: primers for generating high fidelity Cas9, Table S5: sgRNA target sequences for double nicking, Table S6: DNA oligonucleotide sequences used for generation of GFP-turbo mutant and its reparation, Table S7: primers used to detect integration of HIV-1 ∆Env (a) and Tag-BFP (b) transgenes into the human AAVS1 locus.
