# Supplementary material for: Gene Editing in Human Lymphoid Cells: Role for Donor DNA, Type of Genomic Nuclease and Cell Selection Method
Source: Viruses. 2017 Nov 2;9(11):325. doi: 10.3390/v9110325 (PMC5707532; doi:10.3390/v9110325)
Supplement: Supplementary file 1 [file viruses-09-00325-s001.pdf]

SUPPLEMENTARY INFORMATION for

"Gene editing in human lymphoid cells: role for donor DNA, type of genomic nuclease and cell selection method" A. Zotova, E. Lopatukhina, A. Filatov, M. Khaitov, D. Mazurov

Figure S1. Sequence of AAVS1-specific ZFN module. It was designed to facilitate the replacement of zinc finger protein (ZFP) coding regions depending on DNA-binding specificity.

```
 M  D  Y  K  D  D  D  D  K        P  K  K  K  R  K  V
ATGGATTACAAGGATGACGATGACAAGGGCGCCCCCAAGAAGAAGAGGAAGGTGGGCATT
           Flag                                NLS
CACGGGAATTCC
```

ZFP-L

```
 A  A  M  A  E  R  P  F  Q  C  R  I  C  M  R  N  F  S  Y  N
GCCGCTATGGCTGAGAGGCCCTTCCAGTGTCGAATCTGCATGCGTAACTTCAGTtaCaaC

 W  H  L  Q  R  H  I  R  T  H  T  G  E  K  P  F  A  C  D  I
tggcacCTccagCGCCACATCCGCAaCCCACACAGGCGAGAAGCCTTTTGCCTGTGACATT
L binding 1
 C  G  R  K  F  A  R  S  D  H  L  T  T  H  T  K  I  H  T  G
TGTGGGAGGAAGTTTGCCCGCTCCGACcacCTGaccaccCAcACtAAaATcCAtACCGGT
                      L binding 2
 S  Q  K  P  F  Q  C  R  I  C  M  R  N  F  S  H  N  Y  A  R
TCTCAGAAGCCCTTCCAGTGTCGAATCTGtATGaggAACTTtcccacaactacgccCGc
                                              L binding 3
 D  C  H  I  R  T  H  T  G  E  K  P  F  A  C  D  I  C  G  R
gactgccatATtaggACgCAtACAGGCGAGAAGCCTTTTGCCTGTGACATTTGTGGtcga

 K  F  A  Q  N  S  T  R  I  G  H  T  K  I  H  L  R
AAgTTcGCtCAGAACtCCACCCGCatcggcCATACCAAGATACACCTGCGG
           L binding 4
```

Fok-L

```
 G  S  Q  L  V  K  S  E  L  E  E  K  K  S  E  L  R  H  K  L
GGATCCCAGCTGGTGAAGAGCGAGCTGGAGGAGAAGAAGTCCGAGCTGCGGCACAAGCTG

 K  Y  V  P  H  E  Y  I  E  L  I  E  I  A  R  N  S  T  Q  D
AAGTACGTGCCCCACGAGTACATCGAGCTGATCGAGATCGCCAGGAACAGCACCCAGGAC

 R  I  L  E  M  K  V  M  E  F  F  M  K  V  Y  G  Y  R  G  K
CGCATCCTGGAGATGAAGGTGATGGAGTTCTTCATGAAGGTGTACGGCTACAGGGGAAAG

 H  L  G  G  S  R  K  P  D  G  A  I  Y  T  V  G  S  P  I  D
CACCTGGGCGGAAGCAGAAAGCCTGACGGCGCCATCTATACAGTGGGCAGCCCCATCGAT

 Y  G  V  I  V  D  T  K  A  Y  S  G  G  Y  N  L  P  I  G  Q
TACGGCGTGATCGTGGACACAAAGGCCTACAGCGGCGGCTACAATCTGCCTATCGGCCAG

 A  D  E  M  E  R  Y  V  E  E  N  Q  T  R  N  K  H  L  N  P
GCCGACGAGATGGAGAGATACGTGGAGGAGAACCAGACCCGGAATAAGCACCTCAACCCC

 N  E  W  W  K  V  Y  P  S  S  V  T  E  F  K  F  L  F  V  S
AACGAGTGGTGGAAGGTGTACCCTAGCAGCGTGACCGAGTTCAAGTTCCTGTTCGTGAGC
```

G H F K G N Y K A Q L T R L N H I T N C
GGCCACTTCAAGGGCAACTACAAGGCCCAGCTGACCAGGCTGAACCACATCACCAACTGC

N G A V L S V E E L L I G G E M I K A G
AATGGCGCCGTGCTGAGCGTGGAGGAGCTGCTGATCGGCGGCGAGATGATCAAAGCCGGC

T L T L E E V R R K F N N G E I N F
ACCCTGACACTGGAGGAGGTGCGGCGCAAGTTCAACAACGGCGAGATCAACTTC

G G G E G R G S L L T C G D V E E N
AGATCTGGCGGCGGAGAGGGCAGAGGAAGTCTTCTAACATGCGGTGACGTGGAGGAGAAT
2A
P G P R M G Y P Y D V P D Y A P K K
CCCGGCCCTAGGATGGGCTACCCATACGATGTTCCAGATTACGCTGGCGCCCCCAAGAAG
HA NLS
K R K V G I H G L D
AAGAGGAAGGTGGGCATTCACGGTCTAGAC

ZFP-R
A A M A E R P F Q C R I C M R N F S Q S
GCCGCTATGGCTGAGAGGCCCTTCCAGTGTCGAATCTGCATGCGTAACTTCAGTcagtcC

S N L A R H I R T H T G E K P F A C D I
TCCaacCTGgCCCGCCACATCCGCACCCACACAGGCGAGAAGCCTTTTGCCTGTGACATT
R binding 1
C G R K F A R T D Y L V D H T K I H T G
TGTGGGAGGAAGTTTGCCCGCaCCGACtACCTGgtggaCCAcACtAAaATcCAtACCGGT
R binding 2
S Q K P F Q C R I C M R N F S Y N T H L
TCTCAGAAGCCCTTCCAGTGTCGAATCTGtATGaggAACTTttcctaCaaCacCcACCTG
R binding 3
T R H I R T H T G E K P F A C D I C G R
ACCCGCcatATtaggACgCAtACAGGCGAGAAGCCTTTTGCCTGTGACATTTGTGGtcga

K F A Q G Y N L A G H T K I H L R
AAgTTcGCtCAGggCtacaacCTGgCCgGCCATACCAAGATACACCTGCGG
R binding 4

Fok-R
Q L V K S E L E E K K S E L R H K L
GCTAGCCAGCTGGTGAAGAGCGAGCTGGAGGAGAAGAAGTCCGAGCTGCGGCACAAGCTG
NheI
K Y V P H E Y I E L I E I A R N S T Q D
AAGTACGTGCCCCACGAGTACATCGAGCTGATCGAGATCGCCAGGAACAGCACCCAGGAC

R I L E M K V M E F F M K V Y G Y R G K
CGCATCCTGGAGATGAAGGTGATGGAGTTCTTCATGAAGGTGTACGGCTACAGGGGAAAG

```
 H  L  G  G  S  R  K  P  D  G  A  I  Y  T  V  G  S  P  I  D
CACCTGGGCGGAAGCAGAAAGCCTGACGGCGCCATCTATACAGTGGGCAGCCCCATCGAT

 Y  G  V  I  V  D  T  K  A  Y  S  G  G  Y  N  L  P  I  G  Q
TACGGCGTGATCGTGGACACAAAGGCCTACAGCGGCGGCTACAATCTGCCTATCGGCCAG

 A  D  E  M  Q  R  Y  V  K  E  N  Q  T  R  N  K  H  I  N  P
GCCGACGAGATGCAGAGATACGTGAAGGAGAACCAGACCCGGAATAAGCACATCAACCCC

 N  E  W  W  K  V  Y  P  S  S  V  T  E  F  K  F  L  F  V  S
AACGAGTGGTGGAAGGTGTACCCTAGCAGCGTGACCGAGTTCAAGTTCCTGTTCGTGAGC

 G  H  F  K  G  N  Y  K  A  Q  L  T  R  L  N  H  K  T  N  C
GGCCACTTCAAGGGCAACTACAAGGCCCAGCTGACCAGGCTGAACCACAAAACCAACTGC

 N  G  A  V  L  S  V  E  E  L  L  I  G  G  E  M  I  K  A  G
AATGGCGCCGTGCTGAGCGTGGAGGAGCTGCTGATCGGCGGCGAGATGATCAAAGCCGGC

 T  L  T  L  E  E  V  R  R  K  F  N  N  G  E  I  N  F
ACCCTGACACTGGAGGAGGTGCGGCGCAAGTTCAACAACGGCGAGATCAACTTCTGATAA

GGTACC
KpnI
```

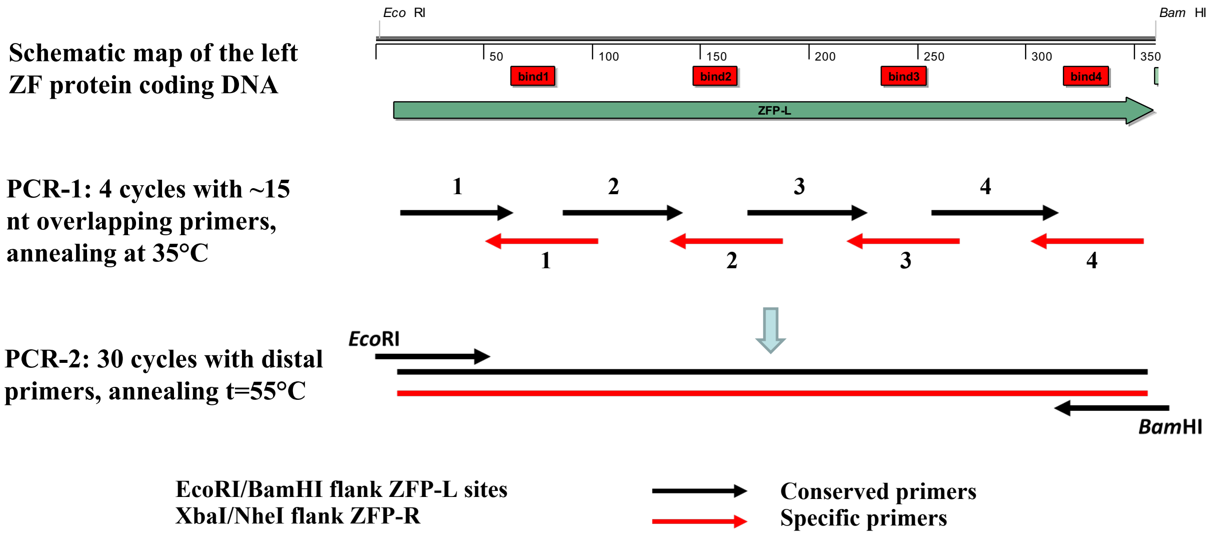

Figure S2. Strategy to assemble ZFP coding DNA using overlapping PCR. Four conserved forward (black arrows) and four specific reverse (red arrows) primers are mixed together and amplified at low annealing temperature. Then pair of end-specific primers which include restriction sites for cloning into pCMV-ZFN plasmid (EcoR I and BamH I for ZFL or Xba I and Nhe I for ZFR) are added to PCR reaction to amplify full length product.

Table S1. The list of primers used to change the specificity of ZFN.

ZF-conserved forward primers (1,2,3,4 are suitable to assemble both left and right ZFP)

1	**GCCGCTATGGCTGAGAGG**CCCTTCCAGTGTCGAATCTG**CATGCGTAACTTCAGT**
2	**CACATCCGCACCCAC**ACAGGCGAGAAGCCTTTTGCCTGTGACATTTGT**GGGAGGAAGTTTGCC**
3	**CACACTAAAATCCAT**ACCGGTTCTCAGAAGCCCTTCCAGTGTCGAATCTG**TATGAGGAACTTTTCC**
4	**CATATTAGGACGCAT**ACAGGCGAGAAGCCTTTTGCCTGTGACATTTGT**GGTCGAAAGTTCGCT**
EcoR I-F	GGAATTCC**GCCGCTATGGCTGAGAGG**
BamH I-R	GGGATCCCCGCAG**GTGTATCTTGGTATG**
Xba I-F	GTCTAGAC**GCCGCTATGGCTGAGAGG**
Nhe I-R	GGCTAGCCCGCAG**GTGTATCTTGGTATG**

AAVS1-specific reverse primers

L1	**GTGGGTGCGGATGTG**GCGCTGGAGGTGCCAGTTGTA**ACTGAAGTTACGCATG**
L2	**GTATGGATTTTAGTGTG**GGTGGTCAGGTGGTCGGAGCG**GGCAAACTTCCTCCC**
L3	**ATGCGTCCTAATATG**GCAGTCGCGGGCGTAGTTGTG**GGAAAAGTTCCTCATA**
L4	CCGCAG**GTGTATCTTGGTATG**GCCGATGCGGGTGGAGTTCTG**AGCGAACTTTCGACC**
R1	**GTGGGTGCGGATGTG**GCGGGCCAGGTTGGAGGACTG**ACTGAAGTTACGCATG**
R2	**GTATGGATTTTAGTGTG**GTCCACCAGGTAGTCGGTGCG**GGCAAACTTCCTCCC**
R3	**ATGCGTCCTAATATG**GCGGGTCAGGTGGGTGTTGTA**GGAAAAGTTCCTCATA**
R4	CCGCAG**GTGTATCTTGGTATG**GCCGGCCAGGTTGTAGCCCTG**AGCGAACTTTCGACC**

CCR5-specific reverse primers

L1	**GTGGGTGCGGATGTG**GCGGGAGAGGTTGGAGCGATC**ACTGAAGTTACGCATG**
L2	**GTATGGATTTTAGTGTG**GGAGTTCAGGTTGGAGGAGAT**GGCAAACTTCCTCCC**
L3	**ATGCGTCCTAATATG**GCGGGCGAGGTTATCGGAGCG**GGAAAAGTTCCTCATA**
L4	CCGCAG**GTGTATCTTGGTATG**GCGGGTGAGGTTGCCGGAGGT**AGCGAACTTTCGACC**
R1	**GTGGGTGCGGATGTG**CACGGACAGGTTGTCGGAGCG**ACTGAAGTTACGCATG**
R2	**GTATGGATTTTAGTGTG**CACCTGTAGGTTGATCTTCTG**GGCAAACTTCCTCCC**
R3	**ATGCGTCCTAATATG**CTCGGACAGCACGTCGGAGCG**GGAAAAGTTCCTCATA**
R4	CCGCAG**GTGTATCTTGGTATG**GGTGGTACGATGGTTGCGCTG**AGCGAACTTTCGACC**

CXCR4-specific reverse primers

L1	**GTGGGTGCGGATGTG**GCGGGAGAGGGCGGAGCGATC**ACTGAAGTTACGCATG**
L2	**GTATGGATTTTAGTGTG**GCGGGTCAGATCATCGGAGCG**GGCAAACTTCCTCCC**
L3	**ATGCGTCCTAATATG**GCGGGCGAGGTTGCCGGACTG**GGAAAAGTTCCTCATA**
L4	CCGCAG**GTGTATCTTGGTATG**GCGGGTGAGGGAGCCGGACTG**AGCGAACTTTCGACC**
R1	**GTGGGTGCGGATGTG**GCGCAGCAGGGAGTCGGAGCG**ACTGAAGTTACGCATG**
R2	**GTATGGATTTTAGTGTG**GGTGGTTAGATGGTCGGAGCG**GGCAAACTTCCTCCC**
R3	**ATGCGTCCTAATATG**GGCGGACAGGGAGTCGGAGCG**GGAAAAGTTCCTCATA**
R4	CCGCAG**GTGTATCTTGGTATG**GCGGGTGAGGTTGGAGCGGTC**AGCGAACTTTCGACC**

Overlapping sequences are highlighted in bold. Restriction sites are underlined

Table S2. Primers for engineering pAAVS1-Δ8.2R donor plasmid

Name	Purpose	Sequence
SacII-F	5'-arm cloning	CCGCGGTGCTTTCTCTGACCAGCATTCTCTCCC
EcoRI-R		GAATTCGGTGACCGTCGACAAGCTTTCTAGAACGCGTCCCCACTGTGGGGTGGAGGGG
EcoRI-F	3'-arm cloning	GAATTCGTTTAAACGATATCCTCGAGGTCATCCTCATCCTGATAAACTGCAAAAGGCTCGAGACTAGGGACAGGATTGGTGACAGAAAAGC
KpnI-R		GGTACCAGAGCAGAGCCAGGAACCCCTGTAG
EcoRI-F	SV40 pA cloning	GAATTCTTAACTTGTTTATTGCAGCTTATAATGGTTAC
XhoI-R		CCTCGAGGGATCCAGACATGATAAGATACATTGATG
Δ8.2R -F	MluI introduction	GTTGGGACGCGTGAATTCGAGCTCGCCCGACATTG
Δ8.2R -R		GCTCGAATTCACGCGTCCCAACAACAACAATTGCATTCATTTTATG

Restriction sites and polylinkers are underlined

Table S3. Primers for engineering pAAVS1-TagBFP donor plasmid

Name	Purpose	Sequence
MluI-F	SV40 pA cloning	CACGCGTCGATCCAGACATGATAAGATAC
XbaI-R		GTCTAGATTAACTTGTTTATTGCAGCTTATAATG
XbaI-F	Tag-BFP cloning	CTCTAGATTAATTAAGCTTGTGCCCCAG
EcoRI-R		CGAATTCGCGGCCGCCATGAGCGAGCTGATTAAGG

Restriction sites and polylinkers are underlined

Table S4. Primers for generating high fidelity Cas9.

Mutation	Primer	Sequence
K848A	forward	CTTTTCTCgcaGATGATTCTATTGATAATAAAGTGTTG
	reverse	CAATAGAATCATCtgcGAGAAAAGACTGGGGCACG
K1003A	forward	CAAAAAATATCCCgcgCTTGAATCTGAATTTGTTTACGG
	reverse	GATTCAAGcgcGGGATATTTTTTGATAAGTGCAGTG
R1060A	forward	GATTCGGAAGgcaCCACTTATCGAAACAAACGGAG
	reverse	GATAAGTGGtgcCTTCCGAATCTCTCCATTGGCG

Low case letters show mutated nucleotides

Table S5. sgRNA target sequences for double nicking.

Ген	Upstream sgRNA	Downstream sgRNA
GFP-turbo	GATGCGGCACTCGATCTCCA	GAACGGCGTGGAGTTCGAGC
CD4	GTCAAAGGTGATCCAAGACT	GGCTCTCCAAGGTCAGGGTC
CD18 (LFA-1)	ACTTCGTGCACTCCTGAGAG	GCCGGGAATGCATCGAGTCG
CD45 (PTPRC)	AGGTGATATTACCCTCAGTC	AAATGACAGCGCTTCCAGAA
CD50 (ICAM-3)	GGGGCGACCCATCCTCCACT	CACGGTGGTGCTGCTTCGCT
CD54 (ICAM-1)	ACCCTCCACCTGGCAGCGTA	TCACCGTGGTGCTGCTCCGT
CD82 (KAI1)	CCCCATCCTAAGCGAGCTGG	CTATGTCTTCATCGGCGTGG

Table S6. DNA oligonucleotide sequences used for generation of GFP-turbo mutant and its reparation.

Name	Purpose	Sequence
C57del-F	C57del mutation	GCATCACCGGACCCTGAACGGCGTGGAG
C57del-R		GTTCAGGGTCCGGTGATGCGGCACTCG
Upstream	sgRNA targeting DNA	GATGCGGCACTCGATCTCCA
Downstream		GAACGGCGTGGAGTTCGAGC
5’-ss	Oligos donor DNA	GAGCGGCCTGCCCGCgATGGAGATCGAGTGCCGCATCACCGG**C**ACCCTGAACGGCGTGGAGTTCGAGCTcGTGGGCGGCGGAGAGG
3’-ss		CCTCTCCGCCGCCCACgAGCTCGAACTCCACGCCGTTCAGGGTGCCGGTGATGCGGCACTCGATCTCCATcGCGGGCAGGCCGCTC
5’-GFP-don	PCR donor generation (500 bp)	ACGGGGATTTCCAAGTCTCC
3’-GFP-don		GATGGCGTGCAGGAAGGG

The nucleotides between which C57 has been deleted are underlined.
The lowcase letters correspond to mutations in protospacer adjacent motif (PAM) with no amino acid changes.

Table S7. Primers used to detect integration of HIV-1 ΔEnv (a) and Tag-BFP (b) transgenes into the human AAVS1 locus

a

Name	Location	Purpose	Product length	Sequence
5’-out-F	5’-AAVS1 out of 5’-HA*	To detect 5’-integration event	1020 bp	GAACTCTGCCCTCTAACGCTG
CMV-R	CMV in transgene			GTAACGCGGAACTCCATATATG
pA-F	SV40pA in transgene	To detect 3’-integration event	1000 bp	CAATGTATCTTATCATGTCTGGATCC
3’-out-F	3’-AAVS1 out of 3’-HA			GGTTCCAGGCCAAGTAGGTG
5’-out-F	5’-AAVS1 out of 5’-HA	To detect intact allele	1843 bp	GAACTCTGCCCTCTAACGCTG
3’-out-R	3’-AAVS1 out of 3’-HA			GGTTCCAGGCCAAGTAGGTG

b

Name	Location	Purpose	Product length	Sequence
5’-out-F	5’-AAVS1 out of 5’-HA*	To detect 5’-integration event	967 bp	GAACTCTGCCCTCTAACGCTG
pA-R	SV40-pA in transgene			CCAAACTCATCAATGTATCTTATC

* HA is homology arm

All primers were designed using NCBI PrimerBLAST web resource; specificity was searched against human both genomic DNA and RNA database

.
